# Post COVID syndrome: A novel challenge and threat to international health

**DOI:** 10.3126/nje.v12i2.46149

**Published:** 2022-06-30

**Authors:** Indrajit Banerjee, Jared Robinson, Alexandra Leclézio, Brijesh Sathian, Indraneel Banerjee

**Affiliations:** 1-3 Sir Seewoosagur Ramgoolam Medical College, Belle Rive, Mauritius; 4 Geriatrics and long term care Department, Rumailah Hospital, Hamad Medical Corporation, Doha, Qatar; 5 Consultant Uro oncologist and Robotic Surgeon, Apollo multi speciality Hospitals, Kolkata, West Bengal, India

**Keywords:** Post-acute COVID-19 syndrome, COVID-19 post-intensive care syndrome, COVID-19, Coronavirus Infections

## Abstract

The global pandemic caused by the SARS-CoV-2 virus has affected every continent worldwide. The novelty of this virus, its mutations and the rapid speed and unprecedented rate at which it has torn through the global community has in turn lead to an innate lack of knowledge and information about the actual disease caused and the severity of the complications associated with COVID-19. The SARS-CoV-2 virus has been infecting individuals since 2019 and now as of 2022 has been circulating for just over 2 years within the global populous. As the number of cases have risen globally over this period (some of which having contracted the virus twice) further endeavours have been undertaken to better understand the pathogenesis and natural progression of the disease. A condition reported in some cases with extended bouts of sickness or symptoms following the initial infection with COVID was labelled “long COVID” towards the earlier phases of the pandemic (in the spring of 2020), but has only recently gained the global media and medical attention due to its affliction of more individuals on a global basis and has thus warranted further investigation. Long COVID is described as a persistent, long-term state of poor health following an infection with COVID-19. The effect of Long COVID is multisystemic in nature with a wide array of signs and symptoms. The most commonly reported clinical features of long COVID are: headaches, myalgia, chest pain, rashes, abdominal pain, shortness of breath, palpitations, anosmia, persistent cough, brain fogs, forgetfulness, depression, insomnia, fatigue and anxiety. This research aims to explore the symptomatology, pathophysiology as well as the treatment and prevention of Long COVID.

## Background

The global pandemic caused by the SARS-CoV-2 virus has affected every continent worldwide. The novelty of this virus, its mutations and the rapid speed and unprecedented rate at which it has torn through the global community has in turn lead to an innate lack of knowledge and information about the actual disease caused and the severity of the complications associated with COVID-19. Superadded to this the long-term ramifications of an infection with COVID-19 are similarly misunderstood and not known due to the lack of prior studies being available to understand the natural history and progression of the disease. On a Global scale, as of the 23rd June 2022, 539,893,858 cases of COVID-19 have been confirmed, 6,324,112 of these being deaths. The SARS-CoV-2 virus has been infecting individuals since 2019 and now as of 2022 has been circulating for just over 2 years within the global populous. As the number of cases have increased globally over this period (some of which having contracted the virus twice) further endeavours have been undertaken to better understand the pathogenesis and natural progression of the disease. It is however clearly evident that a substantial dearth in information surrounding the virus and its long-term effects exists, as a substantial rise in “odd” post COVID symptoms and complications have been reported globally [1,2].

### Progression of the understanding of COVID-19

In the initial phases of the pandemic towards the latter part of 2019 and beginning of 2020, global intelligence and medical knowledge with regards to the SARS-CoV-2 virus and the disease caused by the pathogen was very one dimensional and basic. The general understanding and findings were that an infection with the virus involved mainly the respiratory tract and caused an atypical type of pneumonia, characterized by a ground glass appearance on an x-ray of the chest. The general symptoms of COVID-19 being body ache, cough, fever and in severe cases respiratory failure and ultimately death. The viral genome was sequenced which subsequently revealed further information and allowed for a better understanding of the mechanism of action of the virus. A condition reported in some cases with extended bouts of sickness or symptoms following the initial infection with COVID was labelled “long COVID” in the earlier phases of the pandemic (spring of 2020), but has only recently gained the global media and medical attention due to its affliction of more individuals on a global basis and has thus warranted further investigation [3,4].

### A clinical case definition of post COVID-19 condition by a Delphi consensus (WHO)

The WHO’s case definition of Long COVID: “Occurs in individuals with a history of probable or confirmed SARS CoV-2 infection, usually 3 months from the onset of COVID-19 with symptoms and that last for at least 2 months and cannot be explained by an alternative diagnosis. Common symptoms include fatigue, shortness of breath, cognitive dysfunction but also others and generally have an impact on everyday functioning. Symptoms may be new onset following initial recovery from an acute COVID-19 episode or persist from the initial illness. Symptoms may also fluctuate or relapse over time.” [5].

### Post COVID-19 syndrome / Long COVID

Long COVID has many synonyms and is also known as Post COVID-19 syndrome, Post COVID-19 condition, Post COVID condition, Post COVID syndrome and can ultimately be described as a persistent, long-term state of poor health following an infection with COVID-19. The term “Long COVID” was coined by patients suffering from persistent symptoms in the Spring of 2020 whom did not recover for an extended period (weeks to months) following their initial infection with the SARS-CoV-2 virus. It must be noted that Long COVID can occur in any person previously infected by COVID-19; with the severity of the initial infection bearing no influence on the development of the Post COVID syndrome. It is evident however, that the majority of individuals who contract the COVID-19 virus fully recover within a time frame of four to six weeks without any lingering symptoms and adverse effects to their general health. The World Health Organization (WHO) both recognizes this “Post COVID-19 syndrome” and has developed a case definition for the “Post COVID-19 condition” after a series of conferences held in the month of February 2021 [3,6].

### Symptoms and signs of Long COVID

Individuals whom develop Long COVID suffer from a wide array of signs and symptoms which range from the respiratory system of the patient to their cognitive ability. The most commonly reported clinical features of long COVID are: headaches, myalgia, chest pain, rashes, abdominal pain, shortness of breath, palpitations, anosmia, persistent cough, brain fogs, forgetfulness, depression, insomnia, fatigue and anxiety. A 6-month retrospective cohort study undertaken by Taquet M et al, on 273 618 COVID-19 survivors discovered that 57% of the study populous displayed at least 1 symptom of long COVID during the 6 month follow up period. The symptoms under study being namely: 1) breathing difficulties and or breathlessness, 2) fatigue and or malaise, 3) chest and or throat pain, 4) headaches, 5) GIT symptoms, 6) myalgia, other pain, 7) cognitive symptoms, 8) anxiety and or depression.

The study also revealed that nearly 40% of patients developed long COVID symptoms in the 3–6-month period and did not suffer from any symptoms in the preceding 3 months of the study. It was also noted that Long COVID occurred more in younger adults and marginally more in the female populous [6,7]. A similar study conducted by Hannah E Davis et al, found that more than 91% of the 3762 individuals who participated in the study had a recovery period of greater than 35 weeks. The most frequent symptoms being reported by patients after the 6 month recovery period being fatigue, malaise post exertion and cognitive dysfunction. The majority of patients experienced an increase and relapse of symptoms post exercise or exertion. 1700 of the 3762 patients reported the need for a reduced work schedule as compared to their original schedule after their COVID induced illness [5].

### Long COVID is a multisystemic disorder and involves various organ systems namely the:

- Respiratory system: Cough, chest pain, dyspnoea and abnormal pulmonary radiological findings including fibrosis and interstitial thickening.
- Pancreatic system: Pancreatitis, pancreatic dysfunction and injury with raised amylase and lipase.
- Renal system: Renal dysfunction, impairment, acute renal injury and increased serum creatinine.
- Gastrointestinal system: Nausea, abdominal pain, diarrhoea and prolonged faecal shedding of the virus.
- Cardiovascular system: Vasculitis, coagulopathies, palpitations, carditis, pericarditis, abnormal ECG’s and raised serum troponins.
- Hepatobiliary system: Liver damage, injury, raised hepatic enzymes such as elevated alanine aminotransferases and elevated Aspartate aminotransferases.
- Nervous system “Brain fog”, prolonged fatigue, insomnia, delirium, anxiety, cognitive defects, elevated levels of anxiety and depression.
- Lymphatic system: Atrophic lymphoid follicles in the spleen and lymphopenia with reduced B and T cells.

Not all of the above manifestations will be present in each individual case however prevailing symptoms involving the respiratory and nervous system are very common with fatigue being present in the majority of cases [8,9].

### Ostensible pathophysiology of Long COVID

The precise pathological mechanism by which Long COVID induces its effects on cases, is poorly understood as it effects both the young and aged and does not differentiate between individuals whom have suffered from a severe or mild initial COVID infection. In a 3 month follow up study conducted by Zhao et al, it was evident that 71% of the COVID-19 survivors had abnormalities on pulmonary radiology and 25 % of the cohort had functional impairments [10]. Numerous studies have found similar radiological evidence of pulmonary abnormalities and fibrosis for periods beyond 6 months post the initial infection [11]. COVID is shed through various portals from the human body, from respiratory secretions to faeces. Multiple cases internationally have tested positive for COVID via RT-PCR for months after the initial infection and recovery from disease, thus indicating that the virus continues replicating in certain individual for prolonged periods. The presence of the virus in the body for such extended periods has thus lead scientists to postulate that the continued immune interaction and activation due to the virus may play a role in the development of long COVID [12-13]. An autoimmune basis is thus the most likely cause of Long COVID through disorders of T-cell functions. It has been proposed that SARS-CoV-2 induces an autoimmune reaction through a mechanism known as bystander activation, as in a study conducted by Grigorova M et al, it was found that the amount of T-cells specifically responsive to SARS-CoV-2 represented only a minor percentage of the already activated cells within the patient and thus supports the notion of bystander activation. The presence of high concentrations of infiltrates of CD8+ cells in the tissues and organs of individuals who succumbed to COVID-19 further supports the autoimmune theory [14]. The autoimmune dysfunction of T-cells is closely related to thyroid dysfunction and it has been shown that about 20% of patients who have COVID-19 suffer from thyroid dysfunction, thus such autoimmune reactions occurring in vital organs such as the thyroid may better explain the host of symptoms experienced by patients suffering from Long COVID [15]. The presence of autoantibodies namely: autoantibodies against neutrophils, interferons, connective tissues, certain peptides, antiphospholipid antibodies and auto bodies against the cell nucleus have been identified in individuals suffering from COVID-19 and further supports the theory that Long COVID has an autoimmune causation [16].

### Identification of Long COVID through the use of artificial intelligence

The National Institutes of Health has undertaken the task to identify the characteristics of individuals suffering from Long COVID so as to aid the diagnosis and rapid recognition of the condition. The NIH is taking advantage of the electronic health records provided by the (N3C) National COVID Cohort Collaborative with the aid of artificial intelligence and machine learning so as to better analyse the data from the centralized database. As of May 2022 through the efforts made by the team at the NIH over two hundred thousand (200 000) likely long COVID cases have been identified [17,18].

### Treatment of Long COVID

At this current point in time, no specific pharmacological or surgical treatment for Long COVID exists. Currently the only accepted and recommended treatment for Long COVID is through rehabilitation exercises. The rehabilitation should gradually increase in intensity as the recovery of the patient permits so as to not cause further permanent damage to the organs. A light regimen of aerobic exercise is thus recommended with added breathing exercises to strengthen the respiratory muscles. Apart from the use of physical therapy in combatting Long COVID, cognitive behavioural support as well as psychological support and therapy is indicated [19,20].

### Prevention of Long COVID

Much is still to be studied and understood about the true ramifications, effects and etiopathogenesis of Long COVID. It is however clear that the symptoms experienced by individuals suffering from Long COVID are multisystemic in nature and effect the afflicted across a broad spectrum from reduced income due to a decreased work capacity to the inability to perform various everyday activities. The most suitable and holistic method to prevent Long COVID is simply through the continual strengthening of the COVID-19 immunization schedule on a global scale, with greater effort being placed on mopping up of unvaccinated individuals [18].

## Conclusion

Long COVID is a serious and more notable complication being reported more commonly among individuals who have been infected with the SARS-CoV-2 virus. The ultimate extent to which Long COVID effects the Global community is vast and unquantifiable, however it most certainly impacts the individual and the country alike on both an economical and standard of living basis. It is evident that the Global medical fraternity have made remarkable advancements in the betterment of their knowledge and understanding of the SARS-CoV-2 infection, it is however equally as evident that much is still left to question. The further study and research of Long COVID and its implications is vital, until such studies have been undertaken and new discoveries made the best port of call to prevent long COVID will be through continued immunization of the populous.
